# Editorial: Chemometrics-based Spectroscopy for Pharmaceutical and Biomedical Analysis

**DOI:** 10.3389/fchem.2019.00153

**Published:** 2019-03-27

**Authors:** Hoang Vu Dang, Federico Marini

**Affiliations:** ^1^Department of Analytical Chemistry and Toxicology, Hanoi University of Pharmacy, Hanoi, Vietnam; ^2^Department of Chemistry, Sapienza University of Rome, Rome, Italy

**Keywords:** chemometrics, spectroscopy, Pharmaceutical analysis, biomedical analysis, Wavelet Transform

Spectroscopy is associated with a plethora of different techniques studying the interaction between matter and electromagnetic radiation. Linguistically speaking, the term originates from the Latin word “spectrum” meaning “specter or image/vision,” and the Greek word “σκoπεῖν” meaning “to view or inspect.” In other words, it is concerned with the absorption, emission, or scattering of electromagnetic radiation of different wavelengths, intimately linked to the structure of atoms or molecules under study.

Unambiguously, spectroscopy and optical measurement technologies are of great importance for analysis of chemical composition. Spectroscopic techniques such as UV-Vis and IR are routinely used in laboratories as well as detailed in a great number of pharmacopeia monographs (e.g., United State pharmacopeia, British pharmacopeia and European pharmacopeia) for quality control of excipients, pharmaceutical ingredients and dosage forms. These techniques can offer a rapid, cheap, non-invasive/non-destructive analysis, using both off-line and in-/at-/on-line methodologies. Nevertheless, they are usually limited to the identification and assay by spectral comparison of a test sample against a reference standard. This approach may not be suitably applied to qualitative and quantitative analysis of real-world samples due to the complexity of pharmaceutical and biomedical matrices.

Given the above information, the use of chemometrics in spectroscopy is a must to gain efficiency in accessing spectral data. By definition, chemometrics is the use of mathematical and statistical methods to extract relevant chemical information and to correlate quality parameters or physical properties to analytical data. It means that a chemometrician would refer to the knowledge of chemical and instrumental influences to display in ways allowing chemical interpretation of the system under study (Davies, 2012).

With reference to the most straight-forward explanation of chemometrics, in the present Research Topic, Biancolillo and Marini briefly reviewed the different chemometric approaches applicable in the context of spectroscopy-based pharmaceutical analysis, discussing the unsupervised exploration of the collected data as well as the possibility of building predictive models for both quantitative (calibration) and qualitative (classification) responses.

In another review, Tsenkova et al. described the up-to-date development of multivariate analysis methodology in aquaphotomics, a novel scientific discipline proposed by Tsenkova (2005). In aquaphotomics analysis, an aquaphotome (i.e., a database of water absorbance bands and patterns correlating water structures to their specific functions) is built by using light-water interaction. To deal with such complex multidimensional spectral data, chemometric methods are exploited to remove unwanted influences and extract water absorbance spectral patterns related to the perturbation of interest.

In spectral analysis, wavelets have increasingly shown great potential in chemical studies by being superior to existing signal processing algorithms in noise removal, resolution enhancement, data compression, and chemometric modeling (Chau et al., 2004; Vu Dang, 2014). In practice, multicomponent analysis may not be possible with a traditional UV spectrophotometric method due to spectral overlapping of both active and inactive ingredients of pharmaceutical samples. Majorly based on a series of studies by Dinç and co-workers, the review of Dinç and Yazan clearly detailed the theoretical aspects of wavelet transform (i.e., discrete, continuous, and fractional) and its characteristic application to UV spectroscopic analysis of pharmaceuticals.

For pharmaceutical and biomedical analysis, it is noteworthy that the combination of various spectroscopic techniques is advisable in an effort to scrutinize a complex chemical process. In the present Research Topic, this is truly reflected by the following works: (i) Wani et al. studying interaction of neratinib (an anticancer drug) with bovine serum albumin by using both spectroscopic (spectrofluorometric, UV spectrophotometric and Fourier-transform infrared) and molecular docking approaches, and (ii) Shang et al. designing and synthesizing low-cytotoxicity fluorescent probes based on anthracene derivatives for hydrogen sulfide detection.

Nowadays, the on-going application of vibrational spectroscopy has been increasingly generating an enormous number of papers published in the pharmaceutical and biomedical sciences (Abramczyk et al., 2017; Brody et al., 2017; Bunaciu and Aboul-Enein, 2017). It is thus not surprising that the present Research Topic mainly consists of research articles related to infrared and Raman spectroscopy. For instance, Tian et al. explored the use of chemometrics-based Fourier transform infrared spectroscopy for the investigation of plasma biochemical changes due to acute lead poisoning in a rat model. Ryabchykov et al. investigated a data fusion approach for combining the two most powerful imaging techniques (Raman spectroscopy and matrix-assisted laser desorption/ionization mass spectrometry) to better distinguish different regions within biological samples. Risoluti and Materazzi coupled a miniaturized Near Infrared (NIR) spectrometer to chemometrics as a novel entirely on-site approach for assessment of occupational exposure to hydroxyurea. Zou et al. compiled a NIR spectral library of amoxicillin and potassium clavulanate by using a universal model to resolve sample-collection problems, making quantitative models more specific for Process Analytical Technology control. Dai et al. discovered the linear region of Near Infrared Diffuse Reflectance spectra of different particle sizes by using the Kubelka-Munk theory, to serve as a methodological reference for the performance of prediction models. Chen et al. introduced a novel strategy for the real-time quantification of potassium in infant formula samples, i.e., applying a modified random frog algorithm, adopted in a higher-density discrete wavelet transform domain, to select the most important features of laser-induced breakdown spectra related to potassium. Zhao et al. proved that a pharmaceutical analysis model could be more reliable and robust when its parameters (such as spectral pretreatment, latent factors, variable selection, and calibration methods) were optimized by processing trajectory, possibly integrated into PLS software. Bogomolov et al. suggested a time-domain averaging of spectral variables to improve the accuracy of in-line NIR spectroscopic moisture monitoring in a fluidized bed drying process of pharmaceutical powder. Ma et al. proposed the use of the low-rank estimation method to improve the accuracy and robustness of Partial Least Squares and Support Vector Machine chemometric models being applied to Raman quantitative analysis of pharmaceutical mixtures.

Regarding the instrumentation for vibrational spectroscopy, Chen et al. developed a moving window fast Fourier transform cross-correlation to correct non-linear shifts for synchronization of spectra obtained from different Raman instruments. In another study, Fujiwara and Kano recommended the nearest correlation—based input variable weighting method for efficient and highly-accurate soft-sensor design, which is applicable to NIR data especially when the number of input variables is large.

The idea for this Research Topic originally came from the fact that the state-of-the-art application of chemometrics, in particular wavelet transform, plays a vital role in the field of spectroscopy being unceasingly perfected and matured.

As the title indicates, hopefully, it will serve as a useful guide for spectroscopic analysis in the pharmaceutical and biomedical sciences.

## Author Contributions

HV wrote and FM revised the manuscript.

### Conflict of Interest Statement

The authors declare that the research was conducted in the absence of any commercial or financial relationships that could be construed as a potential conflict of interest.
